# A Successful Pregnancy Despite the Presence of an Intrauterine Fetal Bone Fragment

**DOI:** 10.1155/2019/3064727

**Published:** 2019-10-17

**Authors:** Evangelos Petrakis, Ioannis Chatzipapas, Ioannis K. Papapanagiotou, Panagiotis Fotinopoulos, Panagiota Siemou, Konstantinos Ntzeros, Dimitrios Loutradis

**Affiliations:** 1 Department of Obstetrics and Gynaecology, School of Medicine, National and Kapodistrian University of Athens, Alexandra General Hospital, Lourou and Vasilisis Sofias Avenue, 11528 Athens, Greece

## Abstract

**Objective:**

Intrauterine retention of fetal bones following a termination of a pregnancy is a rare complication. Among the few reported cases in literature, there has been no report describing the birth of a live fetus, despite the presence of an embryonic ossicle within the endometrial cavity.

**Case Report:**

A 28-year-old woman, with an obstetrical history of a miscarriage at the 19^th^ week of gestation, underwent a diagnostic hysteroscopy for evaluation of pelvic pain and infertility, which revealed an intrauterine embryonic ossicle. The patient did not comply with our recommendations to undergo a surgical hysteroscopy. The patient's next visit was during her 9^th^ week of gestation. She was followed up regularly at our Obstetrics Department. Her gestation was uneventful, while an elective caesarean section at the 39^th^ week of gestation was performed.

**Conclusion:**

The present clinical case has demonstrated that achieving childbearing is possible, despite the requirement of removing such bone fragments.

## 1. Introduction

Intrauterine retention of fetal bones following a termination of a pregnancy in the second and third trimester of gestation is correlated with high rates of spontaneous miscarriages. It is a rare complication as it is observed in 0.15% [1]of the women undergoing diagnostic hysteroscopy for investigation of secondary infertility and a history of a recent termination of a pregnancy. Bony fragments usually imitate the role of intrauterine contraceptive devices (ICD) by stimulating the secretion of endometrial prostaglandins, thus resulting in secondary infertility. Once the bones are hysteroscopically removed, and if no coexisting infertility factors are present, normal fertility is restored in most of the cases. Thus, hysteroscopy, in these cases, is both diagnostic and therapeutic. Among the few reported cases in the literature, there has been no report describing the achievement of a pregnancy and the birth of a live fetus, despite the presence of an embryonic ossicle within the endometrial cavity.

## 2. Case Presentation

**Figure 1:**
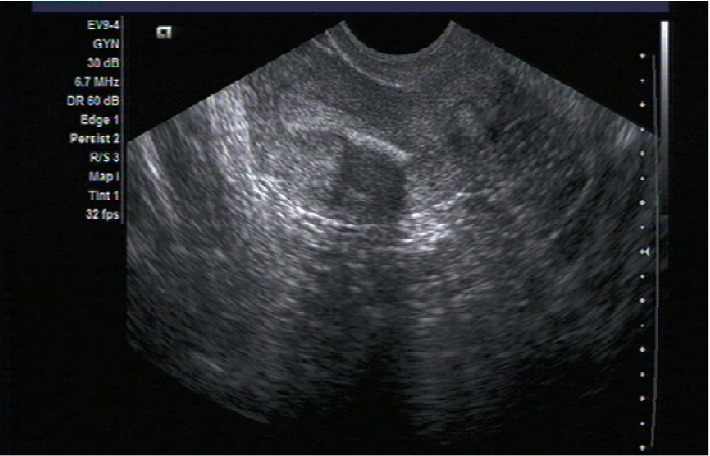
Transvaginal image of the uterus: intrauterine retention of fetal bone.

**Figure 2:**
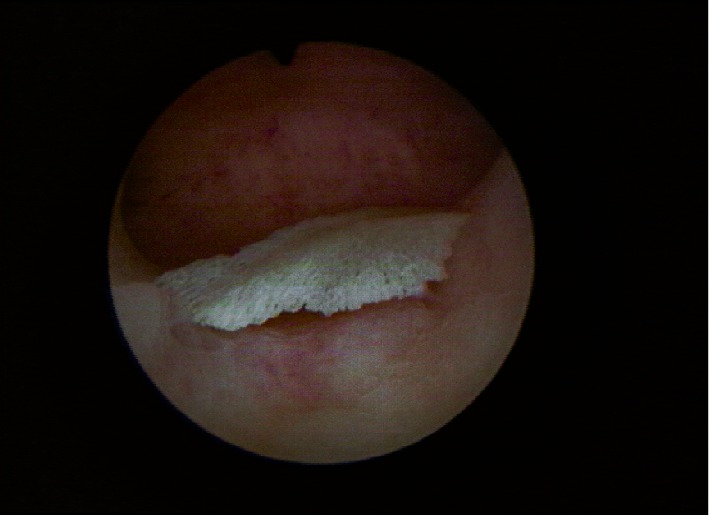
Hysteroscopy: intrauterine retention of fetal bone.

Α 28-year-old woman (G1, P0, A1) visited the Outpatient Gynecological Department of our Hospital complaining about chronic pelvic pain and infertility problems. We evaluated her symptoms and proceeded to the appropriate laboratory and imaging examinations. Ultrasound as well as the mobile diagnostic hysteroscopy performed in the Outpatient Department revealed the presence of an intrauterine embryonic ossicle (Figures 1 and 2). On the other hand, laboratory examinations and patient's vital signs were all normal. Therefore, the patient was scheduled for a surgical hysteroscopy in 2 weeks, with a ultimate goal of removing the fetal bone fragment and restoring the physiological enviroment of the uterus. However, the patient did not comply with our recommendations.

Her obstetric history included a miscarriage at the 19^th^ week of gestation due to placental abruption, one-year prior to her visit to our hospital. The patient declared undergoing an emergency surgical dilatation and curettage, due to heavy bleeding, in order to remove the fetal components.

**Figure 3:**
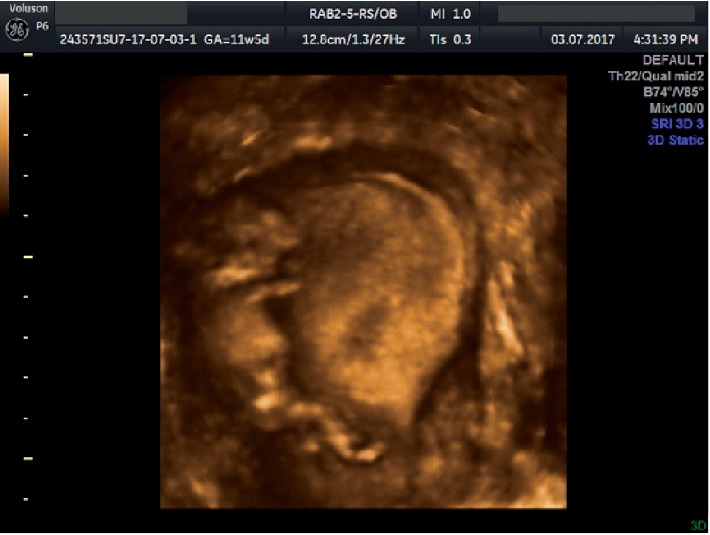
First trimester ultrasound: the concurrent presence of fetus and fetal bone.

Impressively, one year after our initial diagnosis, the patient visited our hospital during her 9^th^ week of gestation. Transvaginal antenatal ultrasound examination revealed the following: fetal movement, crown rump length (CRL) of 26 mm, fetal heart rate of 170 bpm and the intrauterine fetal bone fragment. She was appropriately informed about the risk factors of her gestation and advised to have regular appointments at our Outpatient Obstetrics Department. Obstetric ultrasounds of the 1^st^, 2^nd^and 3^rd^trimesters, as well as the laboratory examinations were all physiological, despite the presence of the intrauterine ossicle (Figure 3).

**Figure 4:**
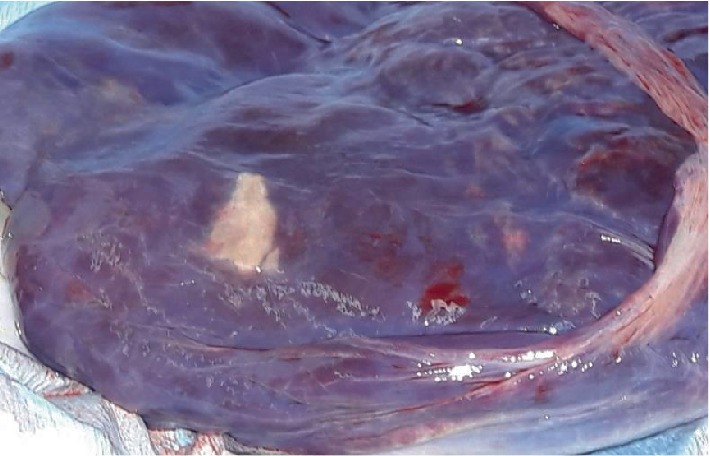
Formed bony tissue on the placenta.

The woman was admitted to the Hospital during her 39^th^ week of gestation and an elective caesarean section was performed, due to her volition. A live and full-fledged fetus was delivered, weighing 3180 kilograms and with an Apgar score of 9 and 10 in the 1^st^ and 5^th^ minute respectively. Following the procedure, we observed formed bony tissue macroscopically in the placenta, therefore the placenta was sent for histopathological analysis (Figure 4). The report from the Pathology Department indicated the presence of placental components and calcium salt deposits as well as the intrauterine rendition of the fetal bone.

## 3. Discussion

Miscarriage is the most common complication of pregnancy in the United States, occurring in 15–20% of clinically recognized pregnancies, or 750,000–1,000,000 cases annually [2]. The vast majority (60%) of miscarriages are due to aneuploidy, whileother established causes of miscarriage include structural abnormalities in the uterus (such as fibroids or a uterine septum), thrombophilias (such as anti-phospholipid syndrome), endocrine disorders (such as hypothyroidism), and autoimmune disorders (such as anti-thyroid antibodies) [2].

Intrauterine retention of fetal bones is a rare complication observed after spontaneous miscarriages or abortions in the second and third trimester in patients undergoing surgical dilatation and curettage. It is found in 0.15% of patients undergoing diagnostic hysteroscopy [1]. However, the percentage of fetal bone fragments removed hysteroscopically in one prospective study, in patients undergoing infertility treatment, reached 11.9% [3]. Such a condition, of fetal bone retention in utero, can result in infertility (72.9%), menstrual abnormalities (30.3%), chronic pelvic pain (10.1%), vaginal bleeding (10.1%), vaginal discharge (4.5%) and dyspareunia (1.1%) [4–6].

Secondary infertility is the most major and frequent complication. Endometrial ossification may cause secondary infertility by three mechanisms: (a) prevention of implantation as a result of obliteration of the uterine cavity (mechanical effect); (b) prevention of implantation as a result of the chronic inflammatory effect of intrauterine bony fragments (intrauterine device-like effect); (c) and direct toxicity of osseous particles on the embryo (embryotoxicity). The extent of uterine involvement is one of the most crucial points determining the effects of endometrial ossification [7].

Ultrasound examination is the gold standard and the most frequently used diagnostic tool, establishing the diagnosis of endometrial ossification. Retained fetal bones appear sonographically as a single or multiple hyperechogenic bands with acoustic shadowing filling the cavity [7]. Transvaginal ultrasound surpasses other diagnostic tools as it is a noninvasive, painless, and relatively low cost method [8]. Furthermore, retained fetal bone fragments can be diagnosed by the presence of filling defects during hysterosalpingography or visualized directly during a hysteroscopy procedure. Although hysterosalpingography and hysteroscopy have traditionally been considered the gold standard methods for uterine evaluation, hysterosalpingography may be unable to detect over 40% of abnormalities of the uterine cavity, while hysteroscopy may overlook bone fragments embedded in the uterine wall [9]. Other diagnostic methods include dilation and curettage, endometrial biopsy, magnetic resonance imaging, and computed tomography [5]. However, histological examination is necessary for the definite diagnosis.

The differential diagnosis of endometrial ossification includes intrauterine contraceptive devices, foreign bodies, calcified submucous fibroids, Asherman syndrome or rare cases such as heterotopic bones. Echogenic endometrium is the most likely finding in these cases [6].

Hysteroscopic resection is the most frequently used method for the removal of bony fragments from the uterine cavity. Surgical treatment with hysteroscopy must be complemented by ultrasonographic guidance to ensure that bone fragments embedded within the myometrium are not missed [9]. In a publication, Dimirtas et al.suggested that dilatation and curettage should be combined with ultrasonography assistance in order to ensure that all abnormal tissue is removed [10]. It is well reported in literature, that in order to achieve high live birth rates and a spontaneous conception, bone fragments should be removed [9].

In conclusion, intrauterine retention of fetal ossicles after a spontaneous miscarriage or termination of pregnancy in the second and third trimester is correlated with secondary infertility. This situation requires the hysteroscopic removal of such bone fragments in order to enable physiological development of the fetus. However, the present clinical case has demonstrated that achieving childbearing is possible, despite the presence of fetal bone fragments within the endometrial cavity.
